# Management of irrational beliefs among pupils: An intervention study using rational emotive behavior therapy

**DOI:** 10.1097/MD.0000000000042648

**Published:** 2025-05-30

**Authors:** Emmanuel Chidobe Okenyi, Anthonia N. Ngwoke, Onyinyechi Igwe, Victor Sunday Ezema, Charity Nneka Anichebe, S’lungile K. Thwala, Christian Sunday Ugwuanyi

**Affiliations:** a Department of Early Childhood and Primary Education, University of Nigeria, Nsukka, Nigeria; b Department of Educational Foundations & Management, University of Eswatini, Kwaluseni, Kingdom of Eswatini; c Department of Science Education, University of Nigeria, Nsukka, Nigeria; d Faculty of Education, University of the Free State, Bloemfontein, South Africa.

**Keywords:** intervention study, irrational beliefs, management, rational emotive behavior therapy

## Abstract

**Background:**

Learners’ convictions and worldviews are crucial driving forces for academic achievements in elementary schools. This is because a child’s conviction determines his/her educational beliefs and dispositions. On this note, this study investigated the effect of rational emotive behavior therapy (REBT) in the management of irrational beliefs (IBs) among pupils. The primary objective was to ascertain the efficacy of REBT in the management of pupils’ IBs.

**Method:**

This research adopted a randomized controlled trail experimental design. A random sampling method was employed to draw a sample of 103 primary school learners, comprising 52 and 51 pupils for treatment and control groups respectively. Besides, G-Power, version 3.1 was used to ensure sample adequacy for the study. Irrational beliefs inventory (IBI) was used for data collection. Repeated measures analysis of variance was used to analyze the data collected. The hypothesis was tested a 0.05 level of significance.

**Results:**

It was revealed that REBT significantly reduced IBs among primary school pupils after the intervention period as well as at the follow-up measure.

**Conclusion:**

REBT is beneficial in the management of IBs among pupils. The study recommends that primary school counselors and psychologists should educate young learners on the negative effects of IBs on their academic pursuits.

## 1. Introduction

Evaluation and assessments are processes through which the extent and magnitude of changes that take place in the learner after instruction is determined. This exercise is effectively carried out in the classroom, which is a place for the exhibition of learners’ intelligence and academic ability.^[1]^ No learner wants to deal with the feelings and the huddles of being branded foolish.^[2]^ Such feelings may lead to some irrational beliefs (IBs) and thoughts. IBs can be aggravated in assessment situations, particularly among elementary school learners.^[3,4]^ Learners in elementary schools are children in the formative age and are sensitive and apprehensive about being denounced or not being complimented.^[5]^ Elementary school learners are in school children, who are within the middle childhood stage of development with the need for tender care and a soft approach to foster rapid and solid physical, social and cognitive development.^[6]^ Elementary school learners otherwise known as pupils are those in the second level of formal education in Nigeria and are between the ages of 6 to 12 years.^[7]^ This group of learners are enthusiastic about their achievements in the school tasks.^[8]^

Achievement refers to any accomplishment that demands courage, dexterity and focus. It is those accomplishments that one takes honor and pride in.^[9]^ Achievement is a reputable outcome of a particular endeavor.^[10]^ While academic achievement is the variable that represent the degree to which a specific educational goal is achieved.^[11–13]^ Academic achievement is the performance outcomes of the student that indicate the level of knowledge acquired after instruction in a particular topic.^[14]^ Academic achievement is, therefore, the expression of the permanent behavioral change observable in elementary school learners after instructions. Motivation is most reliable indicator of academic achievement.

Motivation as an indispensable indicator of academic achievement influences how particular learners learn.^[15]^ According to,^[16]^ evidence abounds to prove that children learn differently, resulting in different types of beliefs among these young learners. These beliefs could be rational, irrational or dysfunctional. Consequently,^[17]^ opined that the individual’s perception and interpretation of his/her academic experiences shape these beliefs. It is obvious that IBs impact negatively on learners’ academic achievement, which depicts early distortion of their perception of the world around them.^[18]^

IBs refer to inflexible, disjointed and one-sided ideas or conclusions that are tenaciously upheld by an individual. IBs are opinions or principles that are unreasoned and inconsistent with reality that are strongly held on to by an individual, even when evidence of their being false abounds.^[19,20]^ According to^[21]^ IBs is a general term that describes a variety of emotional states, from precise cognitive prejudices to a broader class of epistemologically doubtful views, be it superstition, supernatural and pseudoscientific opinions, conspiracy theories or perceptive models (logical vs instinctive reasoning) that are as well uncorroborated self-related convictions. IBs are, therefore, inappropriate solutions developed by an individual in response to some personal challenges. According to Vasile,^[22]^ while confronting events that militate against one’s personal objectives, or an event that conflicts with one’s values, an individual tends to adopt monocratic thinking, irrational, and incorrect conclusions that distort his judgment, the person is said to have IBs. To deal with this type of situation, Albert Ellis in the 1950s developed rational emotive behavior therapy (REBT).

REBT is all about the emotional difficulties being experienced in life by an individual and the resolution strategy the individual develops to deal with the situation. In an attempt to develop a workable solution, the individual may find him/herself in an emotional state he/she can neither express, explain nor define.^[23–25]^ The emotional troubles people go through often stem from the irrational thoughts they develop about themselves, others and their environment.^[4]^ Rezapour,^[26]^ observed that literature indicates that these unreasonable thoughts are characterized by undesirable emotions, which when correlated to some specific events or circumstances are not rational. Such individuals experience such negative thoughts under some specific emotions without being able to discover why. Hence,^[23]^ maintained that such negative thoughts lead to dysfunctional manners that culminate in failures. Consequently, Persons with IBs are more at risk of developing and experiencing some emotional challenges such as anxiety, depression and distress.^[27]^ Therefore, IBs have as a result been formed.^[18]^ According,^[28]^ Ellis used the ABC model to describe the 3 stages of IBs and a possible remedy. Where A stands for adversity or activating events, B stands for the beliefs about events, refereeing to both obvious and underlying thoughts about situations, oneself, and others. While C refers to the consequences, involving the individual’s behavioral and emotional responses. In this model, B is seen as the most important aspect of the solution process since B links A and C in the sense that REBT is concerned with modifying beliefs B for the purpose of obtaining a positive and acceptable decision. In stage B the person arrives at some emotional conclusions as a result of prior incorrect biases. These biases dominate the individual’s emotional state and he/she draws incorrect conclusions due to his/her negative emotional dispositions.

IBs arise when 2 negative situations coexist.^[19]^ The first instance happens when challenges occurring in a person’s life bring about an emotional behavioral disorder, perpetuating itself in the person’s life as IBs. The second scenario arises from an individual’s response to a negative event occasioned by prior IBs. The outcomes of a person’s experiences in addition to negative events taking place, more and new IBs arise in an undesirable environment created by the individual.^[29]^ Therefore, the setting in which the pupil spends his/her school periods, which constitute the most important part of his/her life, becomes favorable for the occurrence of the above situation, signifying that IBs are engrained in the social environment of the pupil.^[30]^ IBs occur in the elementary school system when the learner believes that his incorrect conviction about an academic or school situation is right over the opinion that it is unfitting and that his/her conviction cannot be disputed or countered. Literature abounds depicting REBT as effective in the management of IBs among high school and University students,^[31]^ but there is little or no literature confirming such about elementary school pupils, thereby prompting this study. According to a study conducted by,^[32]^ unrealistic self-expectations which, constitute a breadth of IBs can be mitigated and managed using cognitive behavioral therapy, such as REBT.

In a similar but separate study,^[33]^ argued that with the positive relationship between REBT and academic motivation, REBT would effectively manage learners’ IBs and improve their motivation. REBT is effective in managing and enhancing students’ IBs and academic motivation and has proved very effective in managing junior secondary school students’ attitudes toward mathematics.^[34]^ Again, REBT has been found to have a significant effect on the management and control of high school students’ academic procrastination.^[35]^ REBT was effective in managing the stress and IBs of teachers of special needs children in Nigeria.^[36]^ In another but related study, it was found that REBT was effective in the management of burnout syndrome among university students in Nigeria.^[37]^

In another related but distinct study, it was discovered that REBT was effective in the management of workers’ mental health as well as in the regulation of such workers’ motivation to work.^[38,39]^ REBT has proved very effective in controlling the IBs and behaviors of athletes, as well as enhancing their motivation thereby leading to enhanced performance and success.^[4,40–42]^ Furthermore, REBT is effective in improving students’ academic grit and success.^[43,44]^

From the available empirical studies, the effectiveness of REBT as a cognitive-behavioral therapy (CBT) cannot be exaggerated. However,^[37]^ observed that there is a paucity of literature to establish the effectiveness of REBT among primary school pupils since researchers have focused on other levels of the education system over the years.

### 1.1. Purpose of the study

The main purpose of the study was to determine the efficacy of REBT in the management of pupils’ IBs. Based on the purpose of the study, the researchers hypothesized That REBT has no significance in the management of IBs among pupils.

## 2. Methods

### 2.1. Ethical consideration

The Research and Ethics Committee of the Faculty of Education, University of Nigeria, Nsukka, (RECFEUNN) approved the conduct of this investigation. For eligibility and participation, the researchers distributed informed consent forms to the interested pupils for their parents, guardians as well as teachers to complete and endorse. The investigators were guided by the^[45]^ as well as the^[46]^ ethical principles.

### 2.2. Research design

A randomized control trial (RCT) experimental research design was utilized to execute the study. RCT, according to^[47]^ is a research strategy where the participants are indiscriminately assigned to either experimental or control groups to determine the efficiency or otherwise of a treatment or an intervention. The groups are watched within a given space of time to determine the degree and track of the effect of the intervention. Recently, researchers such as^[35,36,48–50]^ have successfully employed the design in related studies. This design allows the researchers to randomize the participants in the study into experimental and control groups as^[51]^ insist that the design guarantees a greater possibility of equivalence.

### 2.3. Population/sample

**Figure 1.**
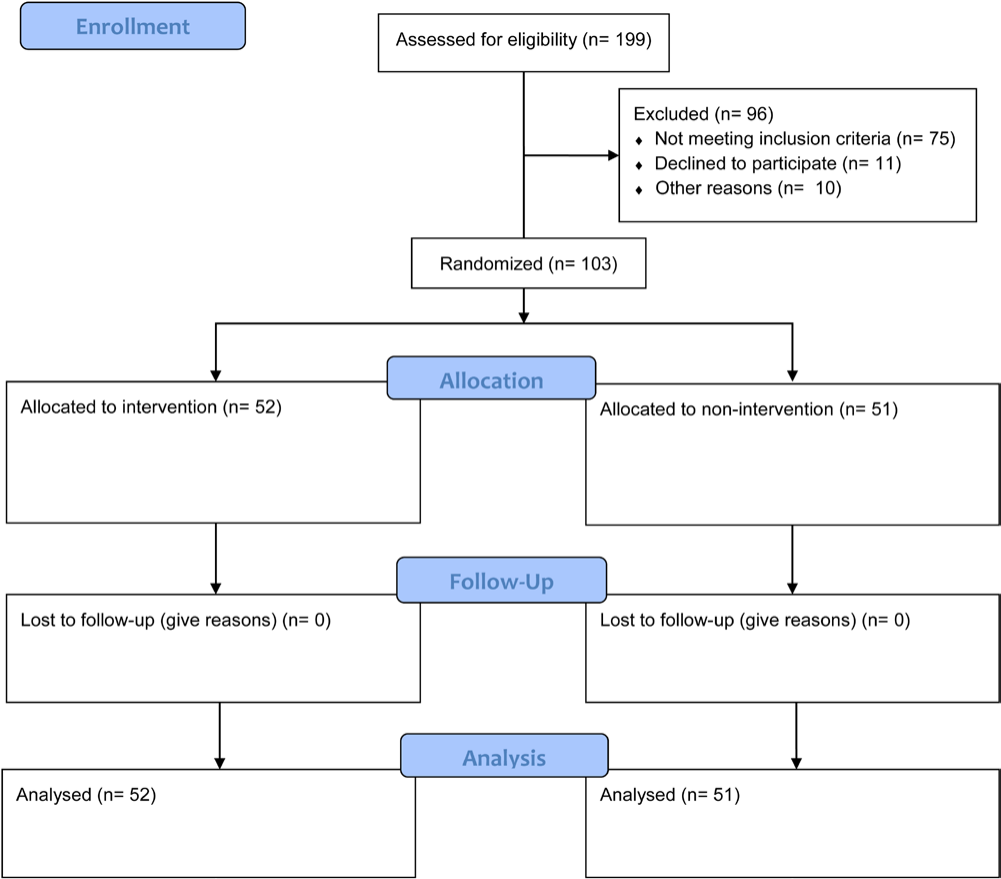
Flow diagram of the participants.

The population of the study is all the primary school children (pupils) within the South Eastern States of Nigeria. The study’s sample size was 103 primary (5) 5 that met the stipulated inclusion conditions. G-Power, version 3.1 within the medium effect of size (f^2^) of 0.15, level of significance at 0.05 and power of 0.84 delivered an acceptable size of 103. Other factors taken into consideration to determine the sample size were groups (2), dependent variables (1), and independent variables (one^[1]^ at 2 levels). This is validated by Faul et al. (2007) who maintain that the power of 0.84 is sufficient to determine an adequate sample size. The entire 199 pupils who showed interest and presented themselves to participate in the program were screened for inclusion based on the researchers’ already set inclusion standards, which include: (a) regularity in school attendance, (b) high in school-related IBs, determined through the use of the Pupils IBs Inventory (PIBI). This was determined by administering the PIBI to the pupils and analysis of the data collected done by the analyst for 8 days. Based on the outcome of the analysis, the researchers penciled down 103 pupils who scaled the inclusion thresholds, and through simple randomization procedure designated them to control and experimental groups. 103 pieces of paper marked control group and experimental group were placed in a container. Thereafter, the pupils/participants were invited to pick, each a paper from the container. Thus, the pupils were randomly designated 51 and 52 pupils to the control and experimental groups respectively as represented in the Figure 1.

## 3. Measures

### 3.1. Questionnaire on demographics

The participants responded to a demographic questionnaire, which revealed their demographic features such as gender, tribe and age as shown in Table 1.

**Table 1:** Demographical profile of the participants.

Demographic features	Treatment group	Control group
Male	23	24
Female	29	27
Total	**52**	**51**
Tribe		
Tiv	2	3
Hausa	3	1
Igbo	42	41
Idoma	1	4
Yoruba	4	2
Total	**52**	**51**
Location		
Urban	46	49
Rural	**4**	2
Total	**52**	**51**

Bold values are just indicating the total number of participants for the treatment and control groups.

### 3.2. PIBI

PIBI was used for data collection. PIBI is a psychometric inventory that was adapted from the Irrational Beliefs Inventory (IBI) that was developed by^[52]^ to measure dysfunctional beliefs as conceptualized in REBT. The IBI poses such inquiries as every individual must be loved and appreciated by all and sundry in everything; Individuals must be accurately competent and achieving in all respects of their lives; It is appalling, atrocious, and disastrous if things/events do not happen as 1 would want them to go; the causes happiness is external and people have no control over their sorrows or purge themselves of negative thinking/feelings. The inventory is made up of 15 items designed on a 5-point Linkert scale. 15 to 75-point scores were assigned to the items on the scale. The scale depicts that increased scores depict more/increased IBs. This implication of this is that the higher the scores the higher the intensity of the IBs.^[53]^

### 3.3. Reliability of the instrument

The reliability index of PIBI was obtained by calculating the internal consistency coefficient as well as the test–retest reliability of the instrument. The item–scale correlations in the inventory are between .51 and .53, and the internal consistency coefficient is .76. After retesting, the reliability coefficient stood at .90, indicating a higher reliability index.

### 3.4. Procedure

In the buildup to the commencement of the intervention program, the researchers published an advertisement calling for interested pupils to indicate interest with the support of their parents, guardians and teachers. In response to this, 199 pupils showed up for the program with their consent forms filed and endorsed by their parents, guardians and teachers. Thus, the researchers administered the PIBI to the 199 pupils who came forward to determine their eligibility based on already determined principles. A pupil who scored 45 points and above on the PIBI was considered eligible for the study. In other words, participants were selected using PIBI based on 45 points benchmark. To this effect, 103 pupils who satisfied the inclusion requirements were selected to participate in the program.

Thereafter, the 103 pupils were randomly assigned to the treatment/intervention and control groups. Each of the groups received detailed and satisfactory explanations of the expectations, objectives and procedures involved in the study. A 4-week intervention program and regular car counseling took place. There was an initial interaction specifically for familiarization to create a free and favorable environment for the implementation of the program. The pupils were made to feel free, safe, and protected by the researchers. The sessions were arranged 3 times a week: Tuesdays, Wednesdays, and Fridays 1 hour each session, for 4 weeks. Within these 4 weeks, the REBT intervention program was administered to the pupils in the treatment group, while those in the control group were given counseling per standard care. The pupils were given the option of personalizing care through the practice of usual care if they so desired. While the treatment group received group therapy, the members of the control group experienced individual counseling sessions for 4 weeks.

At the close of the 4 weeks REBT intervention program, the PIBI was administered to the groups as a post-test measure. Six weeks after the end of the REBT treatment program, a follow-up assessment was carried out using the PIBI to ascertain the quantum of retention of the effect of the REBT treatment on the pupils (participants). It is worth noting that during the recruitment process, the therapist, data analysis, participants, research assistants, and data analysts were all blinded. Data from the pretest, post-test, and follow-up measurements were cleaned and analyzed accordingly.

### 3.5. Control of extraneous variables

A non-differential selection of participants, consistency in assessment measures, avoidance of contact and contamination, removal of selection bias, and elimination of experimental mortality were among the extraneous variables that were found and managed for the study.

By making sure the 2 groups attended their program at distinct places, interaction and contamination were avoided. The PIBI was used at 3 distinct assessment times to ensure consistency in the evaluation measures. With the exception of the intervention program, all other conditions were held constant for the 2 groups in order to prevent non-differential participant selection. In order to eliminate selection bias, participants were asked to select an envelope from a container that contained the letters “E” or “C.” By ensuring that only volunteers who completed the informed consent forms were rewarded and permitted to participate, experimental mortality was prevented.

### 3.6. REBT intervention program

Psychotherapy or CBT is a talking therapy grounded on the interface of emotions. Though this intervention program manual was originally designed to treat depression, it was modified for this study on IBs since IBs and depression are both emotional or mental health issues. Therefore, psychotherapeutic technique that is effective in can also be utilized in managing IBs. After all, the whole idea of CBT is the correction or realignment of one’s negative thoughts, ideas and feelings that arise from IBs. To correct or replace the IBs with more rational and logical ones, the model stresses the significance of pinpointing the ideas and behaviors that underline the IBs, allowing the individual to develop realistic emotional self-control. The treatment sessions in this manual were split into 3 units, each having 3 sessions.

Unit I: how a pupil’s thinking influences his interaction with the environment (teachers, peers, and schoolworks) (sessions 1–3)

This unit provides facts on how a pupil’s (participant’s) thinking and feelings affect his behavior towards the environment. The context and objectives of the remaining sessions are conceptualized in the first session of this unit. Here also, the time, days, and guidelines, as well as the level and magnitude of confidentiality involved in the treatment are well defined. Since the flora and quality of the therapeutic outcomes of the treatment can be affected by the aforementioned variables, the pupils in this unit are made aware of the confidentiality of the treatment. At the beginning of this session, IBs were discussed. Its meaning and how it is perceived by different individuals were highlighted. The counselors/researchers clarified the objectives of this unit. which is to aid the pupils in appreciating how their thoughts and beliefs influence their behaviors and interactions with their environment (teachers, peers, and schoolwork). The emphasis of the remaining 3 sections was on thinking mistakes and school-related IBs of pupils. The pupils (participants) were also. The participants were also instructed on how to counter irrational thoughts and beliefs linked to school environments to manage them. Some drills are employed in between sessions to identify IBs. Again, in these sessions, the pupils were taught the techniques of levitating positive ideas and beliefs and lowering irrational thoughts and beliefs thereby plummeting school-related IBs.

Unit II: How the pupils’ (participants) interaction with the school environment (teachers, peers, school works) affect their motivation to learn (sessions 4–6).

Here the pupils (participants) were helped to connect indicators of IBs with enjoying pleasurable events during sessions 4 to 6. How a pupil’s IBs can hinder him/her from enjoying recreational activities was highlighted. In these sessions, enjoyable recreational activities were discussed and hindrances to participation were noted. The pupils were also exposed to some specific situations that could enable them to decide on objectives that may help reduce school-related IBs. The goals of this unit were to empower the pupils (participants) with more life controls and enlighten them on how to classify choices that will offer them more freedom and possibilities. In these sessions (4–6) the therapists exposed the pupils (participants) experiences on how to develop life skills and inculcated in them the ability to set genuine objectives/goals, and these skills were put into practice. The therapists counseled the pupils on how to set realistic goals and participate in activities that will impact their school experiences positively.

Module III: How the pupils’ relationships (school environment, parents, siblings, society) impact her/his achievement and motivation in their school work (sessions 7–9).

This unit and sessions provided insights into the pupils’ relationships’ impacts on their school experiences (achievement and motivation towards schoolwork) by delineation family and social supports, and how they aid in overcoming school-related challenges and improving academic achievement and motivation. The pupils (participants) were able to acquire the ability to identify and develop their family and social support networks, compliments of these workshops. Themes from the preceding units were integrated into these concluding sessions. The Pupils (participants), together with the therapists (researchers) looked at how the pupils’ thoughts impact how they interact with their environments (school and outside school) their relationships and form their beliefs. Some drills were introduced to enlighten the pupils (participants) on assertive communication skills that will help them develop satisfying and healthy relationships. At the end of the REBT intervention program, the principal themes of each unit were revisited and synchronized to identify the strengths, and the accomplishments were enormous.

### 3.7. Data analysis

SPSS version 25.0 was utilized in carrying out the statistical analysis. The mixed design repeated measures analysis of variance (ANOVA) was employed in the statistical analyses of the data collected. This analytical procedure commonly referred to as a mixed-design analysis of variance model in statistics, provides researchers a platform to accurately compare more than 1 independent group, which their repeated measurements have been taken.

## 4. Results

Table 2 shows that at pretest, the REBT group’s participants had a mean irrational belief score of (M = 49.69, SD = 5.88), while the control group had a mean irrational belief score of (M = 48.94, SD = 5.86). This suggests that both groups’ mean irrational belief scores were comparable at the baseline measure. At the post-test, however, the REBT group’s mean irrational belief score (M = 24.03, SD = 5.02) decreased more than the control group’s (M = 47.88, SD = 5.59). In a similar vein, the REBT group’s mean irrational belief score (M = 23.32, SD = 4.80) decreased more at the follow-up test than the control group’s (M = 47.58, SD = 5.34).

**Table 2:** Irrational belief scores of participants.

Treatment	n	Pretest		Post-test		Follow-up	
		Mean	SD	Mean	SD	Mean	SD
REBT	52	49.69	5.88	24.03	5.02	23.32	4.80
Control	51	48.94	5.86	47.88	5.59	47.58	5.34

**Figure 2.**
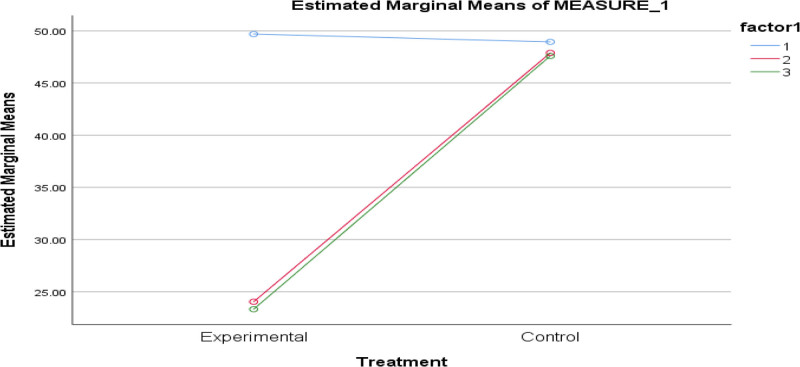
Interaction graph of time and treatment.

Based on Table 3, the irrational belief scores of RBET and control groups were significantly different (between-subjects effect) favoring REBT, *F* (1, 101) = 10798.874, *P* = .000. In addition, there is a significant within-subjects effect due to the different time of measurements, *F* (2, 220) = 300.857, *P* = .000. According to the results, the interaction effect of intervention and time (see Fig. 2) is significant, *F* (2, 202) = 249.694, *P* = .000. According to the effect size of.804, REBT reduced the irrational belief of pupils by 80.4%.

**Table 3:** rm-ANOVA output for the of effect of REBT on dysfunctional attitude of pupils.

Source		Type III sum of squares	df	Mean square	F	Sig.	
		Tests of within-subjects effect					
Time	Sphericity assumed	12726.885	2	6363.442	300.857	.000	.749
Time × treatment	Sphericity assumed	10562.613	2	5281.307	249.694	.000	.712
Error (time)	Sphericity assumed	4272.520	202	21.151			
		Tests of between-subjects effect					
Intercept		500425.523	1	500425.523	10798.874	.000	.991
Treatment		19245.523	1	19245.523	415.307	.000	.804
Error		4680.393	101	46.341			

## 5. Discussion of the findings

This study explored the efficacy of REBT on the management of IBs among pupils. The findings of the study revealed a significant effect of REBT on IBs among pupils. Thus, REBT significantly reduced pupils’ IBs implying that REBT is significant in the management of pupils’ IBs. Due to the wide array of studies on the efficacy of REBT in the management of IBs, the researchers were not surprised at the outcomes of the study. These findings are in line with the findings of^[32]^ who revealed that IBs among high school students can be mitigated and managed using REBT. Furthermore, the results are in line with the findings of,^[33]^ who revealed that REBT is effective in managing learners’ IBs and improving their academic motivation. Again, the findings alined with^[34]^ who revealed that REBT is effective in the management of IBs and improvement of academic motivation among junior secondary school students’ attitudes toward mathematics. More still, the findings confirmed those of^[35–37]^ who pointed out that REBT had significant effects on the management and control of high school students’ academic procrastination, stress and IBs of teachers of special needs children, burnout syndrome among university students in Nigeria. Also, the results of the study are in line with the findings of^[38,39]^ who revealed that REBT was effective in the management of workers’ mental health as well as in the regulation of such workers’ motivation to work. Likewise, comparable research have demonstrated the efficacy of cognitive behavior treatments.^[54–62]^ In a similar vein,^[48]^ found that, in comparison to their counterparts in the waiting-list control group, Nigerian police officers’ work-related stress management was considerably enhanced by rational emotive occupational health coaching.

The outcomes of the study imply that it is imperative to develop and implement cognitive behavioral interventions to manage IBs of pupils. On this note,^[37]^ suggested that the practice of cognitive behavioral group psycho-education program was found effective in reducing IBs in primary schools. Again, the outcomes of this study implies that given pupils’ IBs, critical thinking and problem-solving skills should be inculcated in the pupils to aid them in overcoming such dysfunctional beliefs and becoming better motivated thereby achieving better academic success. To this end,^[43]^ suggested using inquiry-based learning models to assist students in developing critical thinking and problem-solving skills.

## 6. Conclusion and recommendation

The study concluded that REBT is beneficial in the management of IBs among pupils. Thus, proper management of pupils’ irrationals beliefs can be achieved through the use of REBT. Employing REBT in assisting pupils manage their IBs can result to improved achievement and social development of pupils. It is therefore recommended that cognitive behavioral interventions should be established in primary schools to reduce IBs among pupils to help the children develop adequate emotional competence.

## 7. Strengths of the study

The contribution of this work to the field of the management of pupils’ IBs is enormous. Specifically, the management of pupils’ IBs through REBT intervention has been demonstrated by this investigation. This can improve pupils’ self-esteem and enhance their academic achievement and motivation as well as their careers in the long term. Theoretically, the investigation has reinforced the views of REBT theory, which is rooted in classifying counterproductive beliefs and attitudes by challenging their illogicality and replacing them with more positive and progressive ones. Lastly, in terms of policy implications, the investigation has a significant contribution in that there should be an adequate policy plan for the management of pupils’ IBs via the REBT intervention program since the children are the future hope of the nation.

## 8. Limitations of the research

The current study has certain methodological constraints because it is an experimental investigation. First, the results of this study may be limited by the fact that students who are deemed minors were given a 90-minute intervention session after completing the irrational belief inventory in 15 minutes. Second, the effect of REBT may not be adequately controlled for, and it may not be possible to question prior exposure to CBT at baseline. Additionally, this study did not account for the potential moderating effects of gender, age, or location, which could have affected the findings’ generalizability. As a result, it was recommended that future researchers investigate the moderating effects of any of the moderators on the effectiveness of a REBT program on students’ management of irrational belief.

## Author contributions

**Conceptualization:** Emmanuel Chidobe Okenyi, Anthonia N Ngwoke, Onyinyechi Igwe, Victor Sunday Ezema, Charity Nneka Anichebe, S'lungile K Thwala, Christian Sunday Ugwuanyi.

**Data curation:** Christian Sunday Ugwuanyi.

**Formal analysis:** Christian Sunday Ugwuanyi.

**Funding acquisition:** Emmanuel Chidobe Okenyi, Anthonia N Ngwoke, Onyinyechi Igwe, Victor Sunday Ezema, Charity Nneka Anichebe, S'lungile K Thwala, Christian Sunday Ugwuanyi.

**Investigation:** Emmanuel Chidobe Okenyi, Anthonia N Ngwoke, Onyinyechi Igwe, Victor Sunday Ezema, Charity Nneka Anichebe, S'lungile K Thwala, Christian Sunday Ugwuanyi.

**Methodology**: Emmanuel Chidobe Okenyi, Anthonia N Ngwoke, S'lungile K Thwala, Christian Sunday Ugwuanyi.

**Project administration:** Emmanuel Chidobe Okenyi, Anthonia N Ngwoke, Onyinyechi Igwe, Victor Sunday Ezema, Charity Nneka Anichebe, S'lungile K Thwala, Christian Sunday Ugwuanyi.

**Resources:** Emmanuel Chidobe Okenyi, Anthonia N Ngwoke, S'lungile K Thwala, Christian Sunday Ugwuanyi.

**Software:** Christian Sunday Ugwuanyi.

**Supervision:** Emmanuel Chidobe Okenyi, S'lungile K Thwala, Christian Sunday Ugwuanyi.

**Validation:** Christian Sunday Ugwuanyi.

**Visualization:** Christian Sunday Ugwuanyi.

**Writing – original draft:** Emmanuel Chidobe Okenyi, Anthonia N Ngwoke, S'lungile K Thwala, Christian Sunday Ugwuanyi.

**Writing – review & editing:** Christian Sunday Ugwuanyi.
